# Microbial communities on flower surfaces act as signatures of pollinator visitation

**DOI:** 10.1038/srep08695

**Published:** 2015-03-03

**Authors:** Masayuki Ushio, Eri Yamasaki, Hiroyuki Takasu, Atsushi J. Nagano, Shohei Fujinaga, Mie N. Honjo, Mito Ikemoto, Shoko Sakai, Hiroshi Kudoh

**Affiliations:** 1 Center for Ecological Research, Kyoto University, 2-509-3 Hirano, Otsu, Shiga 520-2113, Japan; 2 Institute of Evolutionary Biology and Environmental Studies, University of Zurich, Winterthurerstrasse 190, CH-8057 Zurich, Switzerland; 3 Atmosphere and Ocean Research Institute (AORI), The University of Tokyo, 5-1-5 Kashiwanoha, Kashiwa, Chiba 277-8564, Japan; 4 PRESTO, Japan Science and Technology Agency, Japan

## Abstract

Microbes are easily dispersed from one place to another, and immigrant microbes might contain information about the environments from which they came. We hypothesized that part of the microbial community on a flower's surface is transferred there from insect body surfaces and that this community can provide information to identify potential pollinator insects of that plant. We collected insect samples from the field, and found that an insect individual harbored an average of 12.2 × 10^5^ microbial cells on its surface. A laboratory experiment showed that the microbial community composition on a flower surface changed after contact with an insect, suggesting that microbes are transferred from the insect to the flower. Comparison of the microbial fingerprint approach and direct visual observation under field condition suggested that the microbial community on a flower surface could to some extent indicate the structure of plant–pollinator interactions. In conclusion, species-specific insect microbial communities specific to insect species can be transferred from an insect body to a flower surface, and these microbes can serve as a “fingerprint” of the insect species, especially for large-bodied insects. Dispersal of microbes is a ubiquitous phenomenon that has unexpected and novel applications in many fields and disciplines.

Modern molecular tools allow microbial ecologists to investigate the detailed compositions of microbial communities in various environments, such as soils, aquatic systems, the atmosphere, and human bodies^1^,^2^,^3^,^4^,^5^. High-throughput sequencing provides a cost- and time-effective means of identifying thousands of microbial phylotypes that are present in environmental samples. The technique has revealed that microbial communities are ubiquitous and diverse and that community compositions often distinctly differ among environments^1^,^4^,^6^. More recently, researchers have proposed that microbial communities could be used as tracers that represent host conditions^6^,^7^,^8^.

For example, the microbial community compositions on human palms and fingertips showed distinct differences among individuals^4^, and the individual-specific microbes could move to touched surfaces^7^. Microbial DNA of the individual-specific community can be recovered from the touched surfaces and used to forensically identify the person who touched the object^7^. Another study showed that household members of the same household shared more of their microbiota than individuals from different households^6^. While the microbial community composition is often distinct among environments^1^,^4^,^6^, these studies suggested that microbial cells are relatively easily transferred from one place to another and that the transferred microbes can remain in place for a while.

Transferred microbial cells might contain useful information about the environments from which they came (e.g., human individuals). Therefore, information about the microbial community composition could be regarded as an analog of a human fingerprint on a touched surface^7^; hereafter, we refer to this information as a “microbial fingerprint”. However, practical applications of microbial fingerprints have been limited, with the exceptions described above, although the technique would be a useful tool to detect interactions among orgamisms in ecological studies.

Plant–pollinator interactions would be an interesting system to investigate using the microbial fingerprint technique. Pollinators (e.g., insects) visit flowers to acquire rewards (e.g., nectar) and provide opportunities for plants to disperse their pollen grains, which attach to the pollinators' body surfaces when pollinators visit flowers^9^. Pollinators are required for the production of numerous crops and for the mating success of many wild plants. Thus, they provide critical ecosystem services to humans and help maintain natural plant communities^10^, and therefore a number of researchers has studied plant–pollinator systems. In such studies, identifying the pollinator species provides fundamental information and is most commonly done by direct visual observations^11^. However, this work is laborious and time-consuming, and it requires expertise to identify pollinators in motion.

Microbial communities associated with flowers have been studied^12^, and according to a recent review^12^, fungal communities are most abundantly represented among such studies, followed by bacterial communities. For example, Hererra and his colleagues studied yeasts in floral nectar^13^, and found that nectar-foraging ants transport yeasts to flowers^14^. Furthermore, the transported yeasts induce changes in nectar sugar composition by consuming sugars. Such interactions among flower-insect-yeast have been found in many plant species, and therefore, insect-mediated microbial dispersal may be ubiquitous in nature. However, investigations of microbial communities on insect body surfaces aiming to identify pollinator insect species have not been conducted so far.

In the present study, we hypothesized that the microbial fingerprint technique can provide an alternative to visually identification of potential pollinators of a plant species. To examine whether the microbial fingerprint on an insect body surface could be used to identify potential pollinators, we tested the following hypotheses: *(i)* insect individuals harbor a significant number of microbes on their body surfaces; *(ii)* these microbial cells, the composition of which could be specific to each insect species, are transferred from the insect's body to a flower surface when the insect visits a flower. In addition to these two hypotheses, we compared the results of the microbial fingerprint approach with those of direct visual observation of flower pollinators (i.e., the conventional method) to test the potential usefulness of microbial fingerprinting.

## Results and Discussion

### Microbial cells on the insect body surface

**Figure 1:**
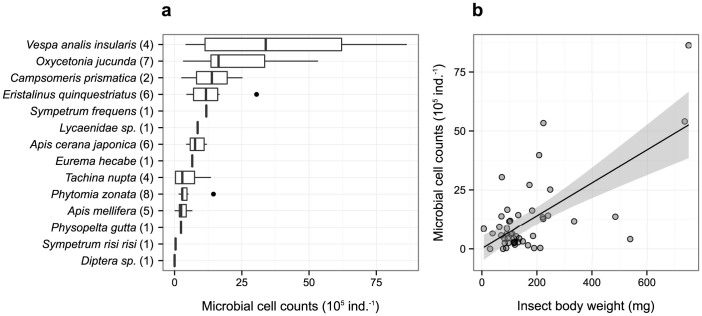
Microbial cell counts and their relationship with insect body weight. (a) Microbial cell counts of collected insect species. Numbers in parentheses indicate how many individuals were analyzed for each insect species. (b) The relationship between insect body weight (i.e., fresh weight) and microbial cell counts. The solid line indicates the linear regression between the two variables. The gray region is the 95% confidence interval of the regression.

Insect samples were collected from October to November, 2012, in Otsu, Shiga Prefecture, Japan (34°58′N, 135°57′E, Alt. *ca.* 150 m). Microbial cells were detached using a sterilized solution and counted under a microscope. Most of the 48 field-collected individuals harbored a sufficient number of microbial cells for detection under the microscope (Fig. 1a and S1). The mean number of detected microbial cells was 12.2 × 10^5^ cells per individual, and the highest mean number was 39.6 × 10^5^ cells from the body surface of a small hornet (*Vespa analis insularis*; Fig. 1a and S1). In addition, we found that the number of microbial cells on an insect's body increased with insect body weight (i.e., fresh weight; Fig. 1b). The greater surface area of larger individuals accommodated more microbial cells than the surface area of smaller insects. These results supported hypothesis *(i)*. An insect with an average body weight generally harbors one million or more microbial cells on its body surface.

### Laboratory contact experiment

To investigate the species-specificity of the microbial community compositions on insect bodies and whether these microbes are transferred to flower surfaces, we conducted a laboratory experiment. Flowers of a pioneer tree species, *Mallotus japonicus*, and its main pollinator insects, carpenter bees (*Xylocopa appendiculata circumvolans*), bumblebees (*Bombus ardens ardens*), and honeybees (*Apis cerana japonica*), were used. This system was chosen because the flowering season and pollination ecology of *M. japonicus* are well documented^15^. By covering buds of male plants before flowering, we obtained male inflorescences that had never been touched by pollinator insects (hereafter referred to as “intact” or “control” flowers). Each pollinator insect was placed in a 2-L plastic container with an intact flower for 3 h, and DNA was extracted from both the insect and plant surfaces. A portion of the 16S small-subunit ribosomal gene was amplified, purified, and sequenced using an IonPGM high-throughput sequencer^16^.

**Figure 2:**
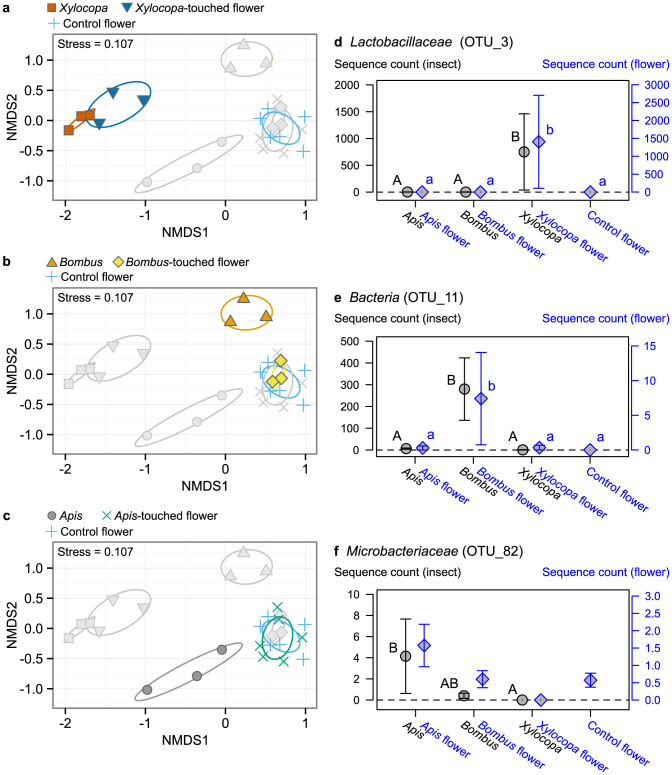
Microbial community compositions of insect and flower surfaces after the laboratory contact experiment. (a–c) NMDS plots of the microbial communities recovered from the insect and flower surfaces. Colors in the plots highlight the results for (a) *Xylocopa*, (b) *Bombus*, and (c) *Apis* and their associated and control flowers. Ovals indicate 95% confidence intervals. (d–f) Sequence counts of unique microbial OTUs for each insect species. Examples of unique microbial OTUs for (d) *Xylocopa*, (e) *Bombus*, and (f) *Apis* are shown. Bars represent standard deviation. Different black capital letters and small blue letters indicate significant differences at *P* < 0.05 for insect and flower samples, respectively. There was no significant difference among the flower treatments in *Apis*-unique OTU (f). For all analyses, raw sequences were globally trimmed to 150 bp, and NMDS using Bray–Curtis dissimilarity was performed. Samples with more than 200 sequences were included in the analysis.

UPARSE processing^17^ of the raw sequences (i.e., 150-bp global trimming and minimum 15 Q-score) identified 207 operational taxonomic units (OTUs) from a the total of 32,620 filtered sequences from 29 samples (Fig. S2a, Table S1). Nonmetric dimensional scaling (NMDS) was performed on the processed sequences using the Bray–Curtis dissimilarity index. The microbial community compositions on insect body surfaces were found to differ significantly among the three insect species (Fig. 2a–c). Flowers touched by carpenter bees showed significant changes in microbial community composition compared with intact flowers (Fig. 2a, *P* < 0.05), while those touched by other insects did not (Fig. 2b, c). Data handling procedures often have significant influence on the results (and biological interpretations) of high-throughput sequencing analysis^17^. However, regardless of the analysis conditions, e.g., 100-, 150-, or 200-bp global trimming, qualitatively similar results were obtained (Fig. S3).

In addition to the overall community composition, microbial OTUs unique to each insect species (i.e., a microbial OTU that was frequently detected from one insect species but almost absent from others; see Supporting Information for detailed identification algorithm) were identified. Among the 207 OTUs, 20 were unique microbial OTUs (Table S2), suggesting that some microbes were transferred from the insect body to the flower surface. A putative *Lactobacillaceae* was frequently detected from carpenter bees but not from the other insect species (Fig. 2d). This *Lactobacillaceae* was also frequently detected from flowers touched by carpenter bees but neither from those contacted by the other insect species nor from the control flowers. This result suggested that microbes on carpenter bees are transferred relatively easily to flower surfaces. This result was in accord with the inferences from the overall community composition. For bumblebees, a unique microbial OTU was a putative *Bacteria* that was detected from bumblebee-touched flowers but not from the other samples (Fig. 2e). Although we did not see a significant difference in overall community composition between the bumblebee-touched and control flowers, the results demonstrated that some microbes could be transferred from bumblebees to the flower surface.

In contrast, for honeybees, although some unique microbial OTUs were identified, there were no significant differences in the sequence counts of the unique microbial OTUs between the honeybee-touched flowers and other flowers (e.g., a putative *Microbacteriaceae*; Fig. 2f). We hypothesized that these differences in the transfer efficiency of microbial cells were attributable to insect body weight. The mean body weight of the carpenter bees used in this experiment was 650 mg, whereas that of the honeybees was less than 100 mg. As the number of microbial cells on an insect's body surface increased with insect body weight (Fig. 1b), smaller insects, such as honeybees, harbor fewer microbes on their body surfaces than larger insects, which might make transfer between the insect and flower difficult to detect. However, considering that more sequences of *Microbacteriaceae* tended to be detected on honeybee-touched flowers than on other samples, it seems possible that more frequent flower visitations by honeybees might alter the microbial community composition of a flower surface. Taken together, our findings indicated that the microbial community composition on flower surfaces can be changed by contact with an individual insect, but the extent of the change depends on insect body weight (or species identity).

### Comparison of the microbial fingerprint approach and direct visual observation

**Figure 3:**
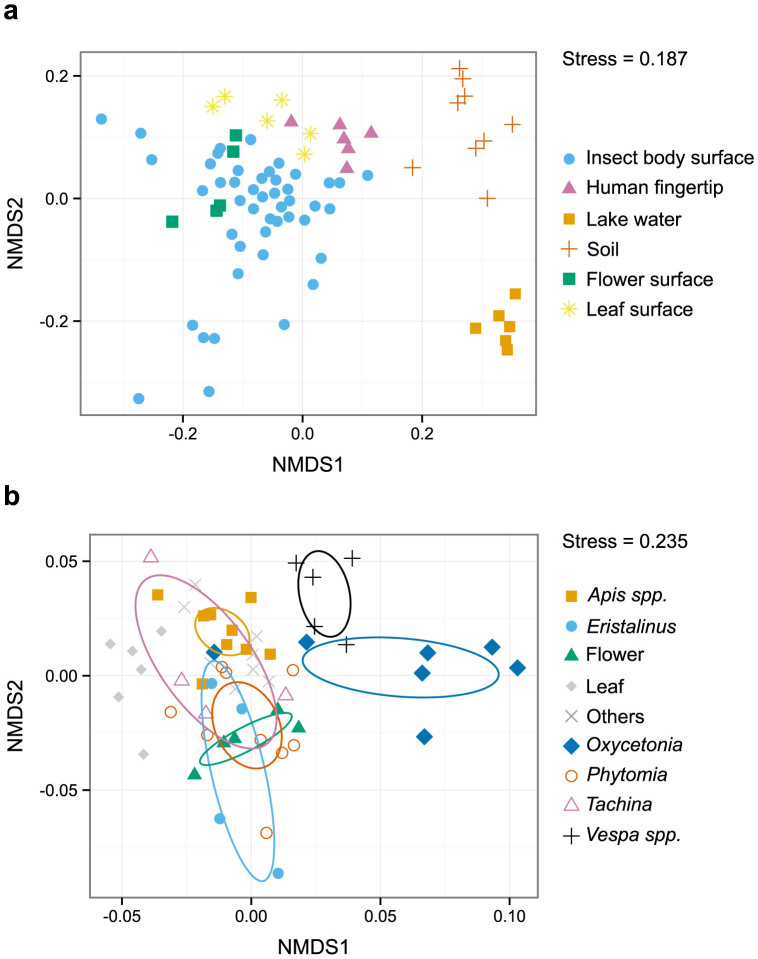
NMDS plots for the microbial communities recovered from insects, flowers, and other environments. (a) Microbial community compositions recovered from insects, leaves, flowers, human fingertips, lake water, and soil. (b) Microbial community composition recovered from insects, leaves, and flowers. Insect species with few replicates are included in the “Others” category. Ovals indicate 95% confidence intervals. For all analyses, raw sequences were globally trimmed to 150 bp, and NMDS using Bray–Curtis dissimilarity was performed. Samples with more than 200 sequences were included in the analysis.

Because the microbial fingerprint approach had the potential to identify insect visitors to flowers, we conducted a field investigation from October to November, 2012, in Otsu, Shiga Prefecture, Japan. We collected insects (the same samples as those used in the microbial cell count experiment), plants, and other environmental samples. We selected tall goldenrods (*Solidago altissima*) as a model plant species for the field research because it is a dominant fall-flowering species in the study area^18^ and because it is a generalist plant, with various insect species visiting its flowers. Environmental samples, including insects, plants, lake water, soil, and human fingertips, were analyzed to compare the microbial community compositions on insect bodies with those of other environments. As a result of UPARSE analysis, 70,829 sequences passed the filtering process and 1,205 OTUs were identified (Fig. S2b–c, Table S3). The microbial communities on insect and plant surfaces had compositions distinct from those of other environmental samples, such as lake water and soil (Fig. 3a). In addition, microbial community compositions on insect bodies showed a degree of species-specificity (Fig. 3b), consistent with the results of the lab experiment (Fig. 2). Furthermore, qualitatively similar results were obtained under different analytical conditions (Fig. S4).

**Figure 4:**
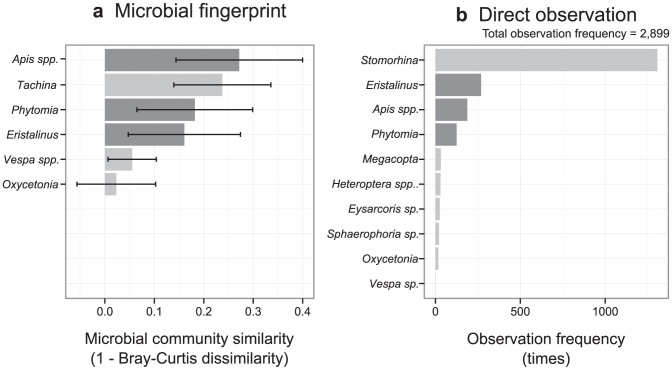
Comparison of the microbial fingerprint approach and direct visual observation. (a) Microbial community similarity between insect and flower samples. Bars indicate standard deviation. (b) Frequency of observation of insects visiting tall goldenrod flowers. The top eight insect taxa (*Stomorhina*, *Eristalinus*, *Apis* spp., *Phytomina*, *Megacopta*, *Heteroptera* spp., *Eysarcoris* sp., and *Sphaerophoria* sp.) and two taxa included in the microbial fingerprinting (*Oxycetonia*, *Vespa* sp.) are shown. Only genus name are shown to conserve space; the full species names are: *Stomorhina* = *Stomorhina obsolete*; *Eristalinus* = *Eristalinus quinquestriatus*; *Apis* spp. = *Apis mellifera* + *Apis cerana japonica*; *Phytomia* = *Phytomia zonata*; *Magacopta* = *Megacopta punctatissima*; *Tachina* = *Tachina nuputa*; *Vespa* spp. = *Vespa analis insularis* + *Vespa mandarinia japonica*, *Oxycetonia* = *Oxycetonia jucunda*. *Eysarcoris* sp., *Sphaerophoria* sp., and *Vespa* sp. could only be identified to the genus level.

To compare the results of microbial fingerprinting with those of direct visual observations, we visually observed insects visiting tall goldenrods during October 2013. For the microbial fingerprint dataset, we calculated the mean values of Bray–Curtis dissimilarity between *Solidago* flowers and insect species. We found that honeybees (*Apis* spp.), a large fly species (*Tachina*), syrphid flies (*Phytomia*), and hoverflies (*Eristalinus*) had relatively similar microbial community compositions to that of *Solidago* flowers (dark gray bars in Fig. 4a), while hornets (*Vespa* spp.) and flower chafers (*Oxycetonia*) had dissimilar ones (Fig. 4a). Direct visual observation suggested that the most frequent flower visitors were a small fly species (*Stomorhina*) (Fig. 4b) that was not collected for the microbial fingerprint approach because of its relatively small body size (≈5 mm). The second most frequent flower visitors were *Eristalinus*, *Apis* spp., and *Phytomia* (dark gray bars in Fig. 4a), in accord with the microbial fingerprint results. *Tachina*, which had microbial community composition similar to that of *Solidago* flowers, was not observed to visit the flowers. Unfortunately, our data cannot explain this lack of visitation by *Tachina*, but we surmised that the differences in study year between the microbial fingerprint study (performed in 2012) and the direct observation (performed in 2013) might be a cause. Indeed, previous studies showed that plant-pollinator interactions often change across time^19^,^20^. Lastly, *Vespa spp.* and *Oxycetonia* were infrequent flower visitors, which was also in accord with the microbial community dataset. In general, despite the inconsistency of the *Tachina* data, these results suggest that the microbial fingerprint approach can clarify the structure of plant–pollinator interactions.

We note that there are several unknown factors involved in the microbial fingerprint approach presented in the study: for example, (1) dispersal of microbes from flower surfaces to insects, (2) quantitative usefulness of the microbial fingerprint approach, and (3) functional and ecological significance of the plant-insect-microbe interactions. First, flowers also harbor microbes on their surface, and the microbial composition is often plant species-specific to some extent^12^. The microbial dispersal from plants to insects was not explicitly examined, but it might influence the community composition of microbes on insect body surfaces. To fully understand the plant-insect-microbe interactions, the microbial dispersal from plants to insects should also be examined. However, our finding that more closely interacting plants and insects (i.e., those that more frequently contacted each other) have similar compositions of their microbial community would not change even if plant-surface microbes have a significant influence on the microbial community composition on insect body surfaces.

Second, as the number of microbes transferred from insects to flowers might depend on the body size of an insect individual (Fig. 1–2), a large number of unique microbes may not necessarily mean that a particular pollinator is a frequent flower visitor. It is possible that an infrequent flower visitor with a large body size might leave more microbial cells on flower surfaces than a frequent flower visitor with a small body size. Therefore, further tests about the quantitative usefulness of the microbial fingerprint approach will be needed. Third, the functional and ecological consequences of the plant-insect-microbe interactions were not examined in this study (because this was not the purpose of this study). However, considering that microbes have many functional roles^14^,^21^, it would be interesting to investigate functional and ecological consequences of the interactions. Our study has provided a base-line dataset for future surveys.

### Conclusions

In the present study, we showed that: *(i)* insect individuals harbor a significant number of microbes on their body surfaces, and *(ii)* some of these microbial cells, the composition of which is somewhat species-specific, are transferred to flower surfaces when the insect visits a flower. Together, our finding showed that insect species-specific surface microbes remaining on a flower surface could be used as “fingerprint” to identify candidate pollinator species for the plant. However, at present, there are several limitations to this technique for reconstructing a plant–pollinator network. For example, the surface microbes of small insects are difficult to detect, so small insect species might not be suitable for microbial fingerprinting. Collecting microbes from body surfaces of many individuals of such species might solve this problem but has not yet been tested. In addition, the species-specificity of insect microbial compositions was less distinct than expected from the results of the human fingertip microbiome^4^,^7^, making the identification of pollinator insects more equivocal.

However, despite the present limitations, the microbial fingerprint approach has potential advantages over the conventional visual observation appproach. For example, microbes could remain on a flower surface for a while, so the microbial community composition might contain cumulative information of flower visitors over a period of time, while direct visual observation detects only a snapshot of flower visitors. Also, the microbial community composition might contain information on flower visitors that are relatively difficult to observe (e.g., those that visit on rainy days or at night). In addition, microbes on an individual pollen grain, if they could be detected, might provide evidence of the pollinator species that carried it. In conclusion, considering recent similar examples^6^,^7^, we emphasize that the transport of microbial cells is a ubiquitous phenomenon that, combined with analyses using modern molecular tools, could be used as a basis to develonovel applications for other fields and disciplines.

## Methods

### Sample collection

Insect and plant samples were collected from October to November, 2012, in Otsu, Shiga, Japan (34°58′N, 135°57′E). Insects were collected with an insect net while wearing sterilized gloves. Our target taxa were flying insects with relatively large body sizes (>0.5–1 cm) because they could be easily handled and were likely to harbor more microbes on their body surfaces than smaller insects. An insect individual collected in the net was carefully placed in a 15-mL sterilized plastic tube without directly contacting the collector's hand. The empty 15-mL tubes were weighed beforehand, and insect fresh weight was calculated by subtracting the weight of the empty tube from that of the tube containing an insect. To avoid microbial contamination among sampling events, the insect net was sterilized with 70% ethanol spray after each collection. Possible microbial contamination of the tube from the air was also tested, but we did not find a significant number of microbial cells in the plastic tube. Flowers and leaves of tall goldenrods (*Solidago altissima*) were collected; this plant is a dominant fall-flowering species in the area^18^. In addition to insects and plants, lake water, soil, and human fingertip samples were collected to determine whether the microbial community compositions on insect and plant surfaces differed from those of the surrounding environment. In total, we collected 48 insect individuals, five flowers (determinate inflorescence), eight individual leaves, nine soil samples, six human fingertip samples, and six lake water samples (Table S3).

### Detachment and counts of microbial cells

Each insect and flower sample was briefly shaken in 5 mL of sterilized 0.1% Tween in 0.15 M NaCl^22^, then microbes were detached by ultrasonic dispersion for 20 s at 50% of maximum power (UR-21P, TOMY, Tokyo, Japan). After the detachment, 1 mL of the solution was stained with 4′,6-deamidino-2-phynylindole (DAPI)^23^, filtered on a polycarbonate filter (0.2 μm pores, ϕ25 mm, Millipore, Billerica, MA, USA) placed on a nitrocellulose filter (0.45 μm pores, ϕ25 mm, Millipore) mounted in a glass holder for the filtration (i.e., two membrane filters were used for one filtration). In addition, 4 mL of the solution was filtered for DNA extraction, and the filter membrane was stored at −20°C until further processing. For each sample, three microscope pictures of DAPI-positive cells with blue excitation (460–490 nm; U-MWB2, Olympus, Tokyo, Japan) were taken at 400× magnification using an epifluorescence microscope (BX60; Olympus) and an attached digital-camera (EOS Kiss X5; Canon, Tokyo, Japan) as described previously^24^. The microscope pictures were then processed with an automated image processing program, the “EBImage” and “biOps” packages of the software R.

### Laboratory contact experiment

To test whether microbes on an insect's body surface moved to a flower surface during visitation, we performed a flower–insect contact experiment. First, at the end of May, 2013, buds of male trees of *Mallotus japonicus* were covered by insect exclusion bags. This species was selected because the flowering season and pollination system have been well described^15^. After flowering (end of June), the exclusion bags were removed and flowers that had never been touched by insects were collected. At the same time, the main insect pollinators of the tree species, carpenter bees (*Xylocopa appendiculata circumvolans*), bumblebees (*Bombus ardens ardens*), and honeybees (*Apis cerana japonica*), were collected. One male inflorescence (*ca*. 10–15 cm long) and one insect individual were immediately placed in a 2-L plastic container, and then the containers were placed on a stable desk in a laboratory for 3 h. Three carpenter bees, five bumblebees, and eight honeybees were used. As a control, seven inflorescence samples were placed in 2-L plastic containers without insects. During the experiment, frequent flower-visiting behaviors were observed. After the experiment, insect individuals and inflorescent samples were immediately frozen and stored at −20°C until detachment, filtration, and DNA extraction.

### DNA extraction, polymerase chain reaction (PCR), and high-throughput sequencing

Each filter membrane was cut into small sections, and DNA was extracted from the sections using a PowerSoil DNA Isolation Kit (MoBio Laboratories, Carlsbad, CA, USA) following the manufacturer's instructions, with an additional incubation step at 65°C for 10 min followed by 3 min of bead beating. Eluted DNAs were stored at −20°C until further processing.

Amplification, purification, and pooling were conducted following Bates et al. (2011)^25^. Briefly, the method includes targeted amplification of a portion of the 16S small-subunit ribosomal gene, triplicate PCR-product pooling (per sample) to mitigate reaction-level PCR biases, and IonPGM high-throughput sequencing (Ion Torrent by Life Technologies, Guilford, CT, USA)^16^. PCR amplification used the primers F515 (5′-GTGCCAGCMGCCGCGGTAA-3′) and R806 (5′-GGACTACVSGGGTA TCTAAT-3′) with IonPGM sequencing adaptors and 6-bp barcode sequences (unique to each individual sample). PCR was performed in 25 μL reactions, each containing 1 μL of 10-μM forward and reverse primers, 10 μL of 5Prime HotMasterMix (Eppendorf-5Prime, Gaithersburg, MD, USA), and 2 μL of extracted DNA as a template. The PCRs were performed as follows: 35 cycles (95°C, 30 s; 50°C, 1 min; 72°C, 1 min) after an initial denaturation at 95°C for 3 min. Triplicate PCR products were pooled and purified using the UltraClean PCR clean-up kit (MoBio Laboratories) and then quantified using Qubit dsDNA HS Assay Kits (Invitrogen, Carlsbad, CA, USA). Equal amounts of PCR product were mixed to produce equivalent sequencing depth from all samples, and the single composite barcoded PCR product was sequenced on an IonPGM at either the Center for Ecological Research in Kyoto University (Shiga, Japan), Macrogen (Tokyo, Japan), or Life Technologies Japan (Tokyo, Japan). The sequence data is deposited in Sequence Read Archive (DRA) of DNA Data Bank of Japan (DDBJ). The accession number is DRA002257 for the submission data.

### Sequence data processing and downstream statistical analysis

UPARSE, which allows accurate OTU identification^17^, was used for quality filtering and OTU clustering of the sequence data. We generally followed the data handling procedure of Edgar^17^ and the website (http://drive5.com/usearch/manual/uparse_cmds.html, Edgar, R., UPARSE Commands, Date of access:19/1/2015). Briefly, the raw FASTQ file was processed by fastq_strip_barcode_relabel2.py script. Then, quality filtering (i.e., global trimming to 150-bp lengths and a minimum Phred score of 15), dereplication, abundance sorting, singleton removal, OTU clustering, and chimera filtering were conducted by following the manual. Taxa were assigned by clidentseq and classignseq commands implemented in Claident^26^ (http://www.claident.org/, Tanabe, A.S., Claident, Date of access:19/1/2015). OTUs identified as chloroplast (i.e., plant-derived DNAs) were excluded from the downstream analysis. UPARSE analyses under different conditions (i.e., global trimming to 100-bp and 200-bp lengths) were also tested, and qualitatively similar results were obtained (Supporting Information).

For the downstream statistical analysis, the statistical environment R^27^ was used. The OTU table generated by the UPARSE and Claident processing was exported using the “phyloseq” package^28^. Nonmetric dimensional scaling (NMDS) using the Bray–Curtis dissimilarity index was performed after the data standardization to visualize the microbial community compositions on flowers, on insects, and in other samples. Unique microbial OTUs were selected under two criteria: 1) the mean value of the sequence counts of a unique microbial OTU detected from a treatment was five-fold larger than the maximum mean value in other treatments, and 2) the coefficient of variation of the sequence counts of a unique microbial OTU was less than 300%. Detailed statistical methods are described in Supporting Information.

### Direct visual observation

Direct visual observation was conducted in October, 2013, in Otsu, Shiga, Japan, where we sampled for the microbial fingerprint study. Flower visitors to *Solidago* flowers were visually counted between 9:00–15:00. For this observation, we established 1 m × 1 m census plots containing 8–10 ramets of *Solidago altissima*. In total, we had 24 census plots across three populations within a 200 m × 110 m area. On sunny or cloudy days, we recorded the number of arthropods found on blooming panicles, including pedicels and peduncles. On each day, some of the census plots were randomly selected and visually observed for a total of 20 min per census plot per day (i.e., 10 min between 9:00–12:00, and 10 min between 13:00–16:00). In total, eight visual observations were conducted for each census plot during the flowering period, a total of 1,920 min (10 min × 8 times × 24 census plots). The flower visitors were identified to the lowest taxonomic level possible in the field. When visual identification was difficult, pictures or representative specimens were taken for identification. In total, 2,899 flower visitors on 231 flowers were recorded during the survey period. For comparison with the microbial fingerprint approach, insects smaller than approximately 4 mm, ants, and spiders were excluded from the analysis.

## Author Contributions

M.U., Y.E., H.T. and S.S. designed research. M.U., Y.E. and H.T. conducted field sampling. M.U., H.T., A.J.N., S.F. and M.N.H. conducted laboratory analysis. A.J.N. and H.K. provided analytical tools. M.I. conducted field observations. M.U. conducted statistical analyses. All authors discussed the results and wrote the manuscript.

## Supplementary Material

Supplementary Information Supporting Infromation

## Notes

The sequence data is deposited in Sequence Read Archive (DRA) of DNA Data Bank of Japan (DDBJ). The accession number is DRA002257 for the submission data.
