# Supplementary material for: Microbial communities on flower surfaces act as signatures of pollinator visitation
Source: Sci Rep. 2015 Mar 3;5:8695. doi: 10.1038/srep08695 (PMC4346974; doi:10.1038/srep08695)
Supplement: Supplementary Information — Supporting Infromation [file srep08695-s1.pdf]

Supporting Information

Microbial communities on flower surfaces act as signatures of pollinator visitation

Masayuki Ushio, Eri Yamasaki, Hiroyuki Takasu, Atsushi J. Nagano, Shohei Fujinaga, Mie N. Honjo, Mito Ikemoto, Shoko Sakai, Hiroshi Kudoh

Contents:

Supplementary Methods.
Sequence data accessibility, sequence data handling procedure, and the unique OTU identification algorithm.

Table S1
Sequence and OTU counts for the lab experiment.

Table S2
Unique microbial OTUs identified in the lab experiment.

Table S3
Sequence and OTU counts for the field research.

Figure S1
An example of collected insects and an image of microbes on an insect body surface.

Figure S2
Taxonomy assigned to the 20 most dominant OTUs using Claident.

Figure S3
NMDS plots for 100-bp, 150-bp, and 200-bp trimming datasets for the lab experiment.

Figure S4
NMDS plots for 100-bp, 150-bp, and 200-bp trimming datasets for the field study.

Supplementary methods

Sequence data accessibility

The sequence data is deposited in Sequence Read Archive (DRA) of DNA Data Bank of Japan (DDBJ). The accession numbers are PRJDB2873 for BioProject, DRA002257 for the submission data, DRX017025–DRX017182 for the experiment data, DRR18836–DRR18993 for the run data, and SAMD00017148–SAMD00017268 for BioSample data.

Sequence handling procedure

UPARSE, which allows accurate OTU identification[1], was used for quality filtering and OTU clustering of the sequence data. We generally followed the data handling procedure described by Edgar[1] and the website (http://drive5.com/usearch/manual/uparse_cmds.html, Edgar, R., UPARSE Commands, Date of access:19/1/2015). The raw FASTQ file was processed by fastq_strip_barcode_relabel2.py script. Then, quality filtering, dereplication, abundance sorting, singleton removal, OTU clustering, and chimera filtering were conducted. Codes for these analyses are described as follows:

1. Stripping
```
python fastq_strip_barcode_relabel2.py data.fq GGACTACVSGGGTATCTAAT tags.fasta Ex > 1_read2.fq
```
2. Quality filtering
```
usearch -fastq_filter 1_read2.fq -fastaout 2_filtered.fa -fastq_truncqual 15 -fastq_trunclen 150 -fastq_qmax 60
```
(Note that the value of the option "–fastq_trunclen" was either of 100, 150, or 200)

3. Dereplication
```
usearch -derep_fulllength 2_filtered.fa -output 3_derep.fa -sizeout
```
4. Abundance sorting
```
usearch -sortbysize 3_derep.fa -output 4_sorted.fa -minsize 2
```
5. OTU clustering
```
usearch -cluster_otus 4_sorted.fa -otus 5_otus1.fa
```
6. Chimera checking
```
usearch -uchime_ref 5_otus1.fa -db gold.fa -strand plus -nonchimeras 6_otus2.fa
```
7. OTU Labeling
```
python fasta_number.py 6_otus2.fa OTU_ > 7_otus.fa
```
8. OTU table creation
```
usearch -usearch_global 2_filtered.fa -db 7_otus.fa -strand plus -id 0.97 -uc 8_map.uc
python uc2otutab.py 8_map.uc > 9_otu_table.txt
```

Taxa were assigned by clidentseq and classignseq commands implemented in Claident[2] (http://www.claident.org/, Tanabe, A.S., Claident, Date of access:19/1/2015) described as follows:

9. Retrieving neighborhood sequences
```
clidentseq blast -strand plus end --blastdb=all_genus --numthreads=24 7_otus.fa otus_claident
```
(7_otus.fa is fasta file generated by the UPARSE analysis)

10. Assigning taxa
```
classigntax --taxdb=all_genus otus_claident tax_claident
```

For the downstream statistical analysis, the free statistical environment R[3] was used. The

OTU table generated by the UPARSE and Claident processing was exported using the "phyloseq" package[4]. Nonmetric dimensional scaling (NMDS) using the Bray–Curtis dissimilarity index was performed to visualize the microbial community composition on flowers, on insects, and in other samples. NMDS using the Bray–Curtis dissimilarity index was chosen because our preliminary analyses suggested that these methods were more suitable for visualizing our dataset compared than principle coordinate analysis using weighted/unweighted UniFrac distance.

Unique OTU identification

Unique microbial OTUs were selected under two criteria: 1) the mean value of the sequence counts of a unique microbial OTU detected from a treatment was five-fold larger than the maximum mean value in other treatments, and 2) the coefficient of variation of the sequence counts of a unique microbial OTU was less than 300%.

First, we calculated mean values and coefficients of variation (i.e., standard deviation divided by mean value) of OTU counts of an insect species (i.e., carpenter bees, bumblebees, and honeybees). We compared the maximum mean value among the three insect species with the second highest mean value. If the maximum mean value was five times greater than the second highest value, the OTU was a candidate unique OTU for the insect species. The candidate OTU was further checked by the second criterion. If the coefficient of variation of the OTU on the candidate insect species was smaller than 300%, we considered the OTU to be unique to the insect species. Under these criteria, a total of 20 OTUs were identified as unique. Among 207 OTUs, 13 were unique to carpenter bees, four to bumblebees, and three to honeybees (Table S2).

References cited in supplementary methods

1. Edgar, R. C. UPARSE: highly accurate OTU sequences from microbial amplicon reads. *Nat. Methods* **10,** 996–998 (2013).
2. Tanabe, A. S. & Toju, H. Two new computational methods for universal DNA barcoding: a benchmark using barcode sequences of bacteria, archaea, animals, fungi, and land plants. *PLoS One* **8,** e76910 (2013).
3. R Core Team. R: A Language and Environment for Statistical Computing. (2014).
4. McMurdie, P. J. & Holmes, S. Phyloseq: An R Package for Reproducible Interactive Analysis and Graphics of Microbiome Census Data. *PLoS One* **8,** e61217 (2013).

Table S1. Sample description for the laboratory experiment

Sample ID	ScientificName	Experiment category	200 bp trimming			150 bp trimming			100 bp trimming		
			Sequence	OTU	(≥ 100 seqs)	Sequence	OTU	(≥ 200 seqs)	Sequence	OTU	(≥ 1000 seqs)
**Control flowers (never touched by insects)**											
No.170	*Mallotus japonicus*	Control flower	308	16	Y	646	30	Y	2911	50	Y
No.171	*Mallotus japonicus*	Control flower	321	21	Y	793	52	Y	2898	82	Y
No.172	*Mallotus japonicus*	Control flower	106	15	Y	353	31	Y	2295	46	Y
No.173	*Mallotus japonicus*	Control flower	12	5	N	46	13	N	941	26	N
No.180	*Mallotus japonicus*	Control flower	227	8	Y	583	20	Y	3573	43	Y
No.181	*Mallotus japonicus*	Control flower	136	20	Y	353	36	Y	4050	72	Y
No.182	*Mallotus japonicus*	Control flower	178	17	Y	477	34	Y	4923	54	Y
**Carpenter bees**											
No.157	*Xylocopa appendiculata circumvolans*	*Xylocopa*	1204	11	Y	2500	15	Y	5075	22	Y
No.159	*Xylocopa appendiculata circumvolans*	*Xylocopa*	1656	15	Y	3772	30	Y	6528	47	Y
No.161	*Xylocopa appendiculata circumvolans*	*Xylocopa*	1228	10	Y	2660	21	Y	4234	33	Y
No.156	*Mallotus japonicus*	*Xylocopa*-touched flower	2616	15	Y	5507	27	Y	9661	48	Y
No.158	*Mallotus japonicus*	*Xylocopa*-touched flower	926	20	Y	2203	43	Y	4070	79	Y
No.160	*Mallotus japonicus*	*Xylocopa*-touched flower	345	22	Y	724	41	Y	1575	67	Y
**Bumblebees**											
No.177	*Bombus ardens ardens*	*Bombus*	135	19	Y	326	34	Y	2869	59	Y
No.179	*Bombus ardens ardens*	*Bombus*	40	19	N	156	61	N	1020	97	Y
No.188	*Bombus ardens ardens*	*Bombus*	298	39	Y	786	73	Y	2032	114	Y
No.190	*Bombus ardens ardens*	*Bombus*	97	22	N	338	55	Y	2226	77	Y
No.202	*Bombus ardens ardens*	*Bombus*	410	5	Y	943	12	Y	1802	33	Y
No.201	*Mallotus japonicus*	Bombus-touched flower	465	41	Y	1513	69	Y	17904	117	Y
No.176	*Mallotus japonicus*	Bombus-touched flower	211	18	Y	684	32	Y	4784	56	Y
No.178	*Mallotus japonicus*	Bombus-touched flower	373	23	Y	912	48	Y	4829	80	Y
No.187	*Mallotus japonicus*	Bombus-touched flower	24	10	N	81	20	N	580	44	N
No.189	*Mallotus japonicus*	Bombus-touched flower	122	16	Y	409	47	Y	3321	67	Y
**Honeybees**											
No.163	*Apis cerana japonica*	*Apis*	8	5	N	32	11	N	101	21	N
No.165	*Apis cerana japonica*	*Apis*	335	14	Y	1093	26	Y	3337	45	Y
No.167	*Apis mellifera*	*Apis*	8	6	N	18	12	N	1089	29	Y
No.169	*Apis cerana japonica*	*Apis*	78	21	N	248	36	Y	2504	53	Y
No.175	*Apis cerana japonica*	*Apis*	38	9	N	102	21	N	387	40	N
No.184	*Apis cerana japonica*	*Apis*	98	10	N	255	20	Y	862	43	N
No.186	*Apis cerana japonica*	*Apis*	39	14	N	108	20	N	319	40	N
No.162	*Mallotus japonicus*	*Apis*-touched flower	28	17	N	104	35	N	330	63	N
No.164	*Mallotus japonicus*	*Apis*-touched flower	306	16	Y	1064	31	Y	4440	52	Y
No.166	*Mallotus japonicus*	*Apis*-touched flower	172	14	Y	337	24	Y	4185	47	Y
No.168	*Mallotus japonicus*	*Apis*-touched flower	294	13	Y	799	23	Y	5104	36	Y
No.174	*Mallotus japonicus*	*Apis*-touched flower	210	18	Y	613	40	Y	1851	74	Y
No.183	*Mallotus japonicus*	*Apis*-touched flower	179	17	Y	507	34	Y	3595	61	Y
No.185	*Mallotus japonicus*	*Apis*-touched flower	384	27	Y	1222	49	Y	3779	79	Y
	Total		13615	103		33267	221		125984	309	
	**Used in the main analysis**		**13145**	**89**		**32620**	**207**		**122464**	**294**	

*"Category" column indicates the group names for the downstream statistical analyses.

Samples highlighted in gray color (indicated by "N) were excluded from the downstream statistical analyses because of the limited number of the high-quality sequence reads.

Table S2. Unique microbial OTU identified.

**OTU ID**	**Insect data**			**Flower data**				**Taxa assigned by Claident**					
	Apis	***Bombus***	***Xylocopa***	***Apis*-touched**	***Bombus*-touche**	***Xylocopa*-touch**	**Control**	**Kingdom**	**phylum**	**class**	**order**	**family**	**genus**
OTU unique to *Xylocopa*													
OTU_3	1.9	1.2	749.3	0.1	0.0	1405.3	0.1	*Bacteria*	*Firmicutes*	*Bacilli*	*Lactobacilla*	*Lactobacillaceae*	
OTU_5	23.9	0.0	1268.0	1.1	0.0	594.7	0.0	*Bacteria*	*Firmicutes*	*Bacilli*	*Lactobacilla*	*Lactobacillaceae*	
OTU_252	0.0	0.0	12.0	0.0	0.0	13.7	0.0	*Bacteria*	*Firmicutes*	*Bacilli*	*Lactobacilla*	*Lactobacillaceae*	
OTU_9	1.1	0.0	432.0	0.4	0.0	228.0	0.0	*Bacteria*					
OTU_165	0.0	0.0	19.3	0.0	0.0	39.7	0.0	*Bacteria*	*Firmicutes*	*Bacilli*	*Lactobacillales*		
OTU_314	0.0	0.0	83.3	0.0	0.0	29.7	0.0	*Bacteria*	*Firmicutes*	*Bacilli*	*Lactobacillales*		
OTU_30	0.0	0.0	16.7	0.1	0.0	71.3	0.0	Unknown					
OTU_41	0.0	0.0	42.7	0.1	0.0	47.0	0.0	*Bacteria*	*Bacteroidete*	*Flavobacter*	*Flavobacter*	*Flavobacteriaceae*	
OTU_260	0.0	0.0	15.0	0.0	0.0	20.3	0.0	*Bacteria*	*Firmicutes*				
OTU_462	0.1	0.0	52.0	0.0	0.0	14.7	0.0	*Bacteria*	*Firmicutes*				
OTU_152	0.0	0.0	26.3	0.0	0.0	3.3	0.0	*Bacteria*	*Firmicutes*	*Bacilli*	*Lactobacillales*		
OTU_18	0.1	0.0	108.0	0.6	0.0	79.3	0.0	*Bacteria*	*Actinobacte*	*Actinobacte*	*Bifidobacter*	*Bifidobacteriaceae*	
OTU_91	0.4	0.0	12.3	0.0	0.0	6.3	0.0	*Bacteria*	*Actinobacte*	*Actinobacte*	*Bifidobacter*	*Bifidobacter*	Bifidobacterium
OTU unique to *Bombus*													
OTU_11	6.3	280.2	0.7	0.3	7.4	0.3	0.0	*Bacteria*					
OTU_8	0.6	14.4	0.0	44.6	27.6	0.0	224.3	*Bacteria*	*Proteobacte*	*Gammaprot*	*Pseudomon*	*Pseudomon*	Pseudomonas
OTU_238	1.1	6.6	0.0	8.7	33.6	0.0	11.6	*Bacteria*	*Proteobacte*	*Gammaprot*	*Pseudomon*	*Pseudomon*	Pseudomonas
OTU_118	0.0	7.2	0.0	5.1	8.6	1.7	3.7	*Bacteria*	*Proteobacte*	*Alphaproteo*	*Rhizobiales*		
OTU unique to *Apis*													
OTU_143	9.7	0.0	0.0	0.0	0.0	0.0	0.0	*Bacteria*	*Proteobacte*	*Betaproteobacteria*			
OTU_10	7.1	1.2	0.3	0.0	0.0	2.3	0.0	*Bacteria*	*Proteobacte*	*Gammaproteobacteria*			
OTU_82	4.1	0.4	0.0	1.6	0.6	0.0	0.6	*Bacteria*	*Actinobacte*	*Actinobacte*	*Actinomycet*	*Microbacteriaceae*	

Numbers indicate the mean value of sequence count of each category

Table S3. Sample description for natural samples

Sample ID	Sample name	Category*	Sampling date	200 bp global trimming			150 bp global trimming			100 bp global trimming		
				Sequence	OTU	≥ 100 bp	Sequence	OTU	≥ 200 bp	Sequence	OTU	≥ 1000 bp
Insect and plant samples												
NO.51	*Apis cerana japonica*	*Apis spp.*	2012/10/24	100	22	Y	517	88	Y	1555	149	Y
NO.55	*Apis cerana japonica*	*Apis spp.*	2012/10/24	370	30	Y	1387	81	Y	3994	106	Y
NO.52	*Apis cerana japonica*	*Apis spp.*	2012/10/24	68	15	N	330	55	Y	961	77	Y
NO.37	*Apis cerana japonica*	*Apis spp.*	2012/10/22	95	16	N	901	42	Y	2231	76	Y
NO.24	*Apis cerana japonica*	*Apis spp.*	2012/10/22	11	5	N	45	17	N	160	45	N
NO.40	*Apis cerana japonica*	*Apis spp.*	2012/10/22	5	3	N	24	13	N	112	42	N
NO.49	*Apis mellifera*	*Apis spp.*	2012/10/24	150	42	Y	705	157	Y	2169	284	Y
NO.38	*Apis mellifera*	*Apis spp.*	2012/10/22	181	26	Y	574	65	Y	1459	127	Y
NO.25	*Apis mellifera*	*Apis spp.*	2012/10/22	53	17	N	537	76	Y	1462	117	Y
NO.42	*Apis mellifera*	*Apis spp.*	2012/10/22	50	21	N	307	78	Y	910	111	Y
NO.20	*Apis mellifera*	*Apis spp.*	2012/10/22	208	21	Y	1087	75	Y	3165	104	Y
NO.48	*Campsomeris prismatica*	Others	2012/10/24	82	27	N	486	93	Y	1620	159	Y
NO.29	*Campsomeris prismatica*	Others	2012/10/22	257	30	Y	1183	71	Y	3427	78	Y
NO.27	*Diptera sp.*	Others	2012/10/22	273	30	Y	1356	86	Y	3360	125	Y
NO.54	*Eristalinus quinquestriatus*	*Eristalinus*	2012/10/24	242	32	Y	803	61	Y	2514	92	Y
NO.44	*Eristalinus quinquestriatus*	*Eristalinus*	2012/10/24	52	12	N	250	52	Y	899	83	N
NO.43	*Eristalinus quinquestriatus*	*Eristalinus*	2012/10/22	61	5	N	215	15	Y	734	38	N
NO.39	*Eristalinus quinquestriatus*	*Eristalinus*	2012/10/22	46	9	N	173	22	N	696	34	N
NO.59	*Eristalinus quinquestriatus*	*Eristalinus*	2012/11/2	28	3	N	155	16	N	696	26	N
NO.23	*Eristalinus quinquestriatus*	*Eristalinus*	2012/10/22	93	5	N	307	10	Y	802	23	N
NO.16	*Eurema hecabe*	Others	2012/10/22	213	24	Y	993	95	Y	2984	137	Y
NO.15	*Lycaenidae sp.*	Others	2012/10/22	175	24	Y	671	58	Y	1892	86	Y
NO.56	*Oxycetonia jucunda*	*Oxycetonia*	2012/11/2	328	32	Y	1510	92	Y	3490	119	Y
NO.06	*Oxycetonia jucunda*	*Oxycetonia*	2012/10/11	406	33	Y	1540	71	Y	3879	95	Y
NO.07	*Oxycetonia jucunda*	*Oxycetonia*	2012/10/11	573	17	Y	1819	34	Y	4574	53	Y
NO.47	*Oxycetonia jucunda*	*Oxycetonia*	2012/10/24	384	15	Y	1421	33	Y	3544	50	Y
NO.05	*Oxycetonia jucunda*	*Oxycetonia*	2012/10/11	335	17	Y	1280	33	Y	2918	49	Y
NO.08	*Oxycetonia jucunda*	*Oxycetonia*	2012/10/11	427	15	Y	1901	33	Y	4211	46	Y
NO.46	*Oxycetonia jucunda*	*Oxycetonia*	2012/10/24	124	9	Y	770	17	Y	2109	34	Y
NO.57	*Physopelta gutta*	Others	2012/11/2	107	25	Y	554	44	Y	2239	71	Y
NO.04	*Phytomia zonata*	*Phytomia*	2012/10/11	631	43	Y	2180	89	Y	4877	119	Y
NO.53	*Phytomia zonata*	*Phytomia*	2012/10/24	243	32	Y	1016	72	Y	2549	106	Y
NO.58	*Phytomia zonata*	*Phytomia*	2012/11/2	198	27	Y	913	76	Y	2626	104	Y
NO.03	*Phytomia zonata*	*Phytomia*	2012/10/11	367	25	Y	1713	55	Y	5015	95	Y
NO.45	*Phytomia zonata*	*Phytomia*	2012/10/24	111	19	Y	366	38	Y	1357	74	Y
NO.01	*Phytomia zonata*	*Phytomia*	2012/10/11	905	18	Y	2649	46	Y	6086	72	Y
NO.26	*Phytomia zonata*	*Phytomia*	2012/10/22	81	14	N	258	41	Y	776	64	Y
NO.02	*Phytomia zonata*	*Phytomia*	2012/10/11	781	22	Y	2364	43	Y	4971	59	Y
NO.19	*Phytomia zonata*	*Phytomia*	2012/10/22	101	7	Y	293	15	Y	1267	22	Y
NO.21	*Phytomia zonata*	*Phytomia*	2012/10/22	50	4	N	139	8	N	540	12	N
NO.32	*Solidago altissima* (Flower)	Flower	2012/10/22	90	18	N	353	42	Y	957	66	N
NO.36	*Solidago altissima* (Flower)	Flower	2012/10/22	231	19	Y	865	44	Y	2229	65	Y
NO.34	*Solidago altissima* (Flower)	Flower	2012/10/22	144	16	Y	526	37	Y	1476	53	Y
NO.33	*Solidago altissima* (Flower)	Flower	2012/10/22	94	13	N	396	32	Y	1217	52	Y
NO.35	*Solidago altissima* (Flower)	Flower	2012/10/22	53	11	N	206	18	Y	758	29	N
NO.68	*Solidago altissima* (Leaf)	Leaf	2012/11/12	111	32	Y	535	89	Y	1370	144	Y
NO.62	*Solidago altissima* (Leaf)	Leaf	2012/11/12	63	15	N	313	58	Y	924	138	N
NO.63	*Solidago altissima* (Leaf)	Leaf	2012/11/12	153	29	Y	596	71	Y	1604	136	Y
NO.67	*Solidago altissima* (Leaf)	Leaf	2012/11/12	125	20	Y	487	55	Y	1327	86	Y
NO.66	*Solidago altissima* (Leaf)	Leaf	2012/11/12	67	18	N	304	33	Y	891	73	N
NO.64	*Solidago altissima* (Leaf)	Leaf	2012/11/12	56	12	N	236	30	Y	667	58	N
NO.70	*Solidago altissima* (Leaf)	Leaf	2012/11/12	2	1	N	32	9	N	86	10	N
NO.17	*Sympetrum frequens*	Others	2012/10/22	523	31	Y	2402	69	Y	7062	84	Y
NO.18	*Sympetrum risi risi*	Others	2012/10/22	175	37	Y	700	85	Y	2000	106	Y
NO.61	*Tachina nupta*	*Tachina*	2012/11/12	93	38	N	420	142	Y	1376	300	Y
NO.65	*Tachina nupta*	*Tachina*	2012/11/12	249	20	Y	742	51	Y	1889	96	Y
NO.30	*Tachina nupta*	*Tachina*	2012/10/22	136	24	Y	688	62	Y	1960	82	Y
NO.31	*Tachina nupta*	*Tachina*	2012/10/22	42	10	N	211	35	Y	689	58	N
NO.10	*Vespa analis insularis*	*Vespa spp.*	2012/10/17	110	29	Y	835	86	Y	2108	129	Y
NO.13	*Vespa analis insularis*	*Vespa spp.*	2012/10/17	78	22	N	387	76	Y	1268	105	Y
NO.11	*Vespa analis insularis*	*Vespa spp.*	2012/10/17	52	27	N	311	71	Y	915	102	N
NO.12	*Vespa analis insularis*	*Vespa spp.*	2012/10/17	116	23	Y	624	65	Y	1676	78	Y
NO.22	*Vespa mandarinia japonica*	*Vespa spp.*	2012/10/22	153	19	Y	960	52	Y	2263	69	Y
Environmental samples												
NO.72	Soil sample	Soil	2012/11/16	123	45	Y	760	198	Y	2645	365	Y
NO.73	Soil sample	Soil	2012/11/16	125	61	Y	656	237	Y	2168	422	Y
NO.74	Soil sample	Soil	2012/11/16	145	60	Y	930	255	Y	3070	457	Y
NO.75	Soil sample	Soil	2012/11/16	165	51	Y	932	237	Y	3071	408	Y
NO.76	Soil sample	Soil	2012/11/16	216	61	Y	1272	195	Y	4050	263	Y
NO.77	Soil sample	Soil	2012/11/16	120	38	Y	881	166	Y	3068	239	Y
NO.78	Soil sample	Soil	2012/11/16	209	39	Y	1255	162	Y	4553	239	Y
NO.79	Soil sample	Soil	2012/11/16	154	46	Y	1062	140	Y	3665	217	Y
NO.80	Soil sample	Soil	2012/11/16	62	31	N	532	164	Y	2285	358	Y
NO.81	Human fingertip	Fingertip	2012/11/16	194	27	Y	1072	79	Y	3615	102	Y
NO.82	Human fingertip	Fingertip	2012/11/16	182	31	Y	1067	130	Y	3207	176	Y
NO.83	Human fingertip	Fingertip	2012/11/16	366	54	Y	1850	132	Y	5341	163	Y
NO.84	Human fingertip	Fingertip	2012/11/16	258	32	Y	1458	92	Y	4166	137	Y
NO.85	Human fingertip	Fingertip	2012/11/16	229	41	Y	1241	111	Y	3717	148	Y
NO.86	Human fingertip	Fingertip	2012/11/16	336	42	Y	1559	132	Y	4429	217	Y
NO.91	Lake water (Lake Biwa)	Lake water	N.A.	283	26	Y	983	82	Y	3531	122	Y
NO.92	Lake water (Lake Biwa)	Lake water	N.A.	232	38	Y	916	104	Y	2823	170	Y
NO.93	Lake water (Lake Biwa)	Lake water	N.A.	268	23	Y	1174	83	Y	4396	137	Y
NO.94	Lake water (Lake Biwa)	Lake water	N.A.	312	34	Y	1031	100	Y	3065	199	Y
NO.95	Lake water (Lake Biwa)	Lake water	N.A.	251	32	Y	1075	91	Y	3521	136	Y
NO.96	Lake water (Lake Biwa)	Lake water	N.A.	210	29	Y	870	86	Y	2994	126	Y
	Total			16321	440		71397	1230		204922	1566	
	**Used in the main analysis**			**14794**	**411**		**70829**	**1205**		**191749**	**1526**	

*"Category" column indicates the group names for the downstream statistical analyses.

Samples highlighted in gray color (indicated by "N) were excluded from the downstream statistical analyses because of the limited number of the high-quality sequence reads.

Note that the sample NO.66 (*Solidago* leaf sample) was excluded from the analysis because severe contamination (highly distinctive community composition from other samples) was suspected.

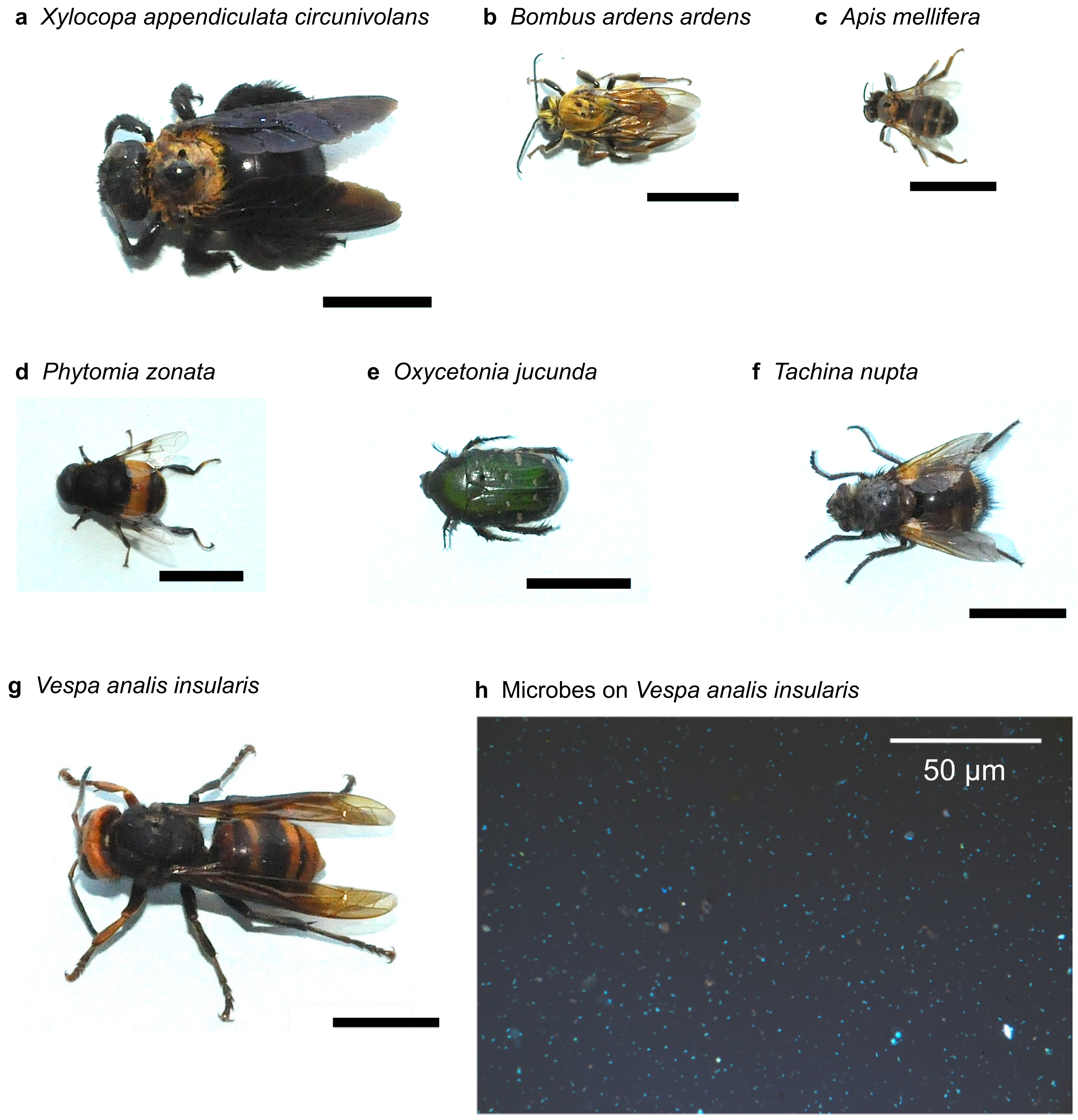

Figure S1.
Examples of collected insects (a–g) and a microscopic image of stained microbes on *Vespa analis insularis* (h). Black bars in the insect pictures indicate 1 cm (a–g). Bright blue cells in the microscopic image were microbes stained with DAPI (see Methods). All photos were taken by M. Ushio.

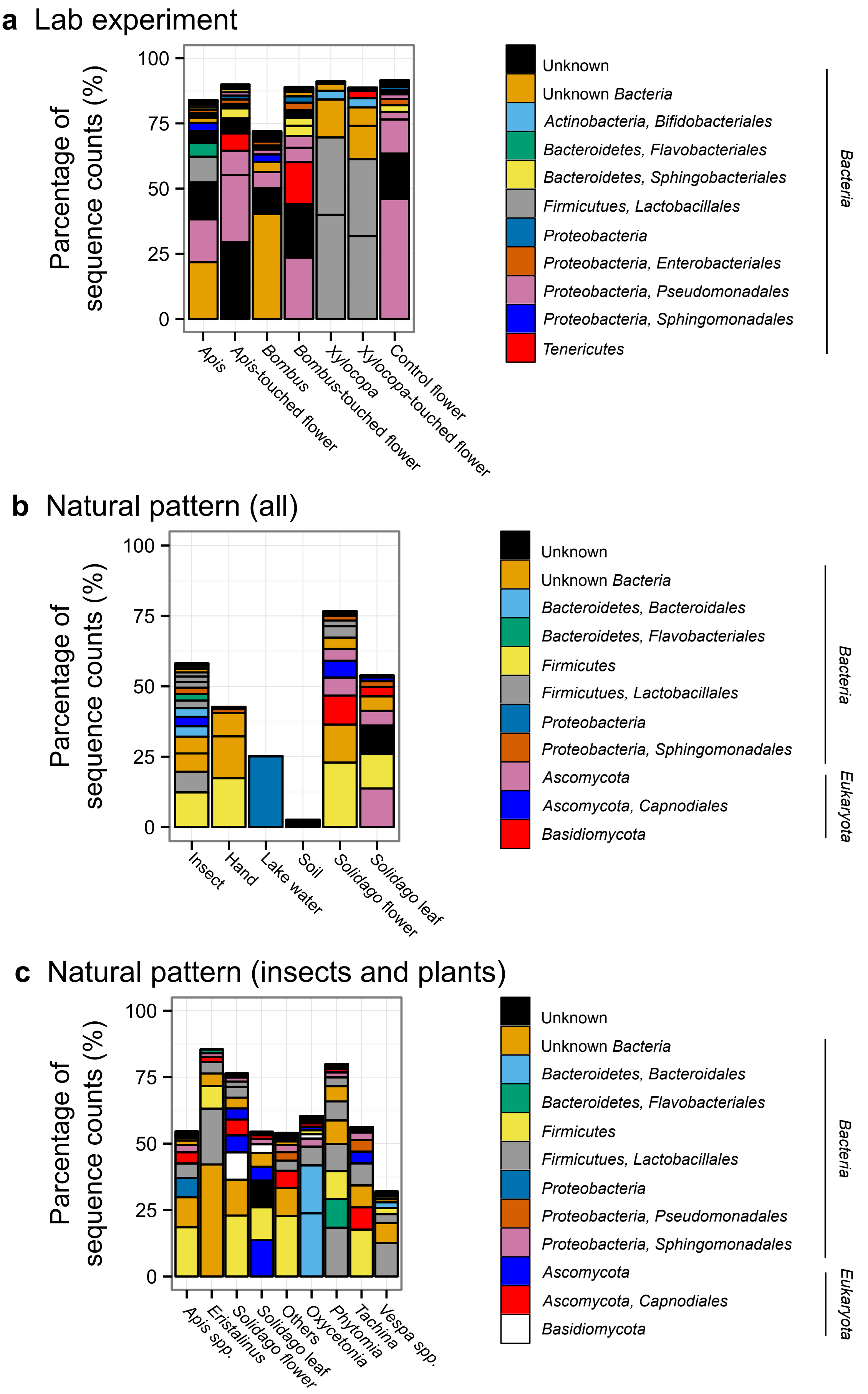

Figure S2

Summary of assigned taxonomy for dominant OTUs for (a) lab contacting experiment, (b) natural pattern (all sample included), and (c) natural pattern (only insect and plant samples). Only the 20 most dominat OTUs are shown. Bar colors were assinged based on the OTU's phylum and order. Taxomony assignment was performed using Claident[2].

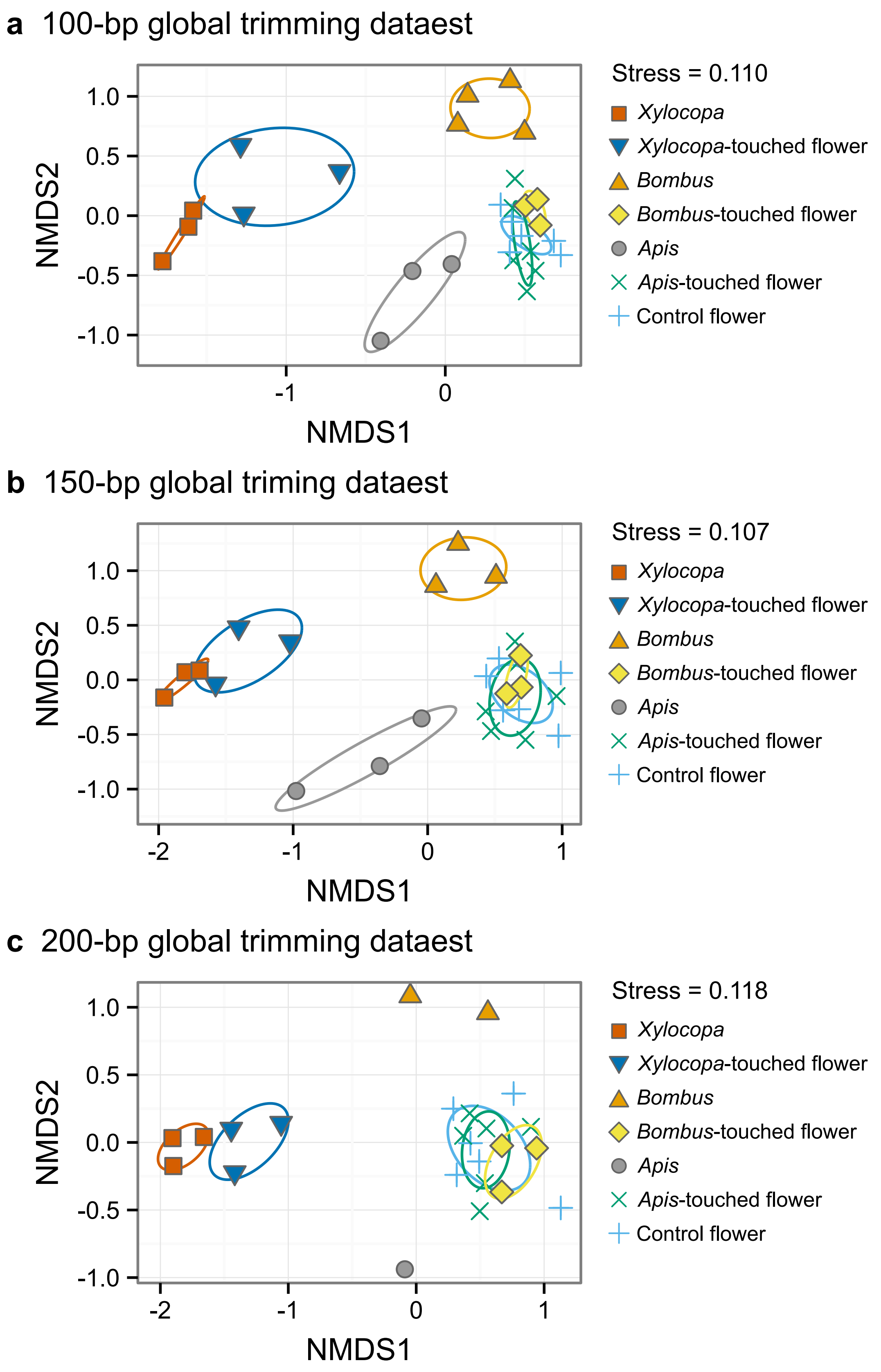

Figure S3

Microbial community composition of the insect and flower surfaces in the laboratory contacting experiment, analyzed using sequence datasets with (a)100-bp, (b)150-bp, and (c) 200-bp global trimming. The NMDS plot of the 150-bp dataset is identical to the NMDS plot in the Figure 2 of the main text, but replicated here for direct compariton.

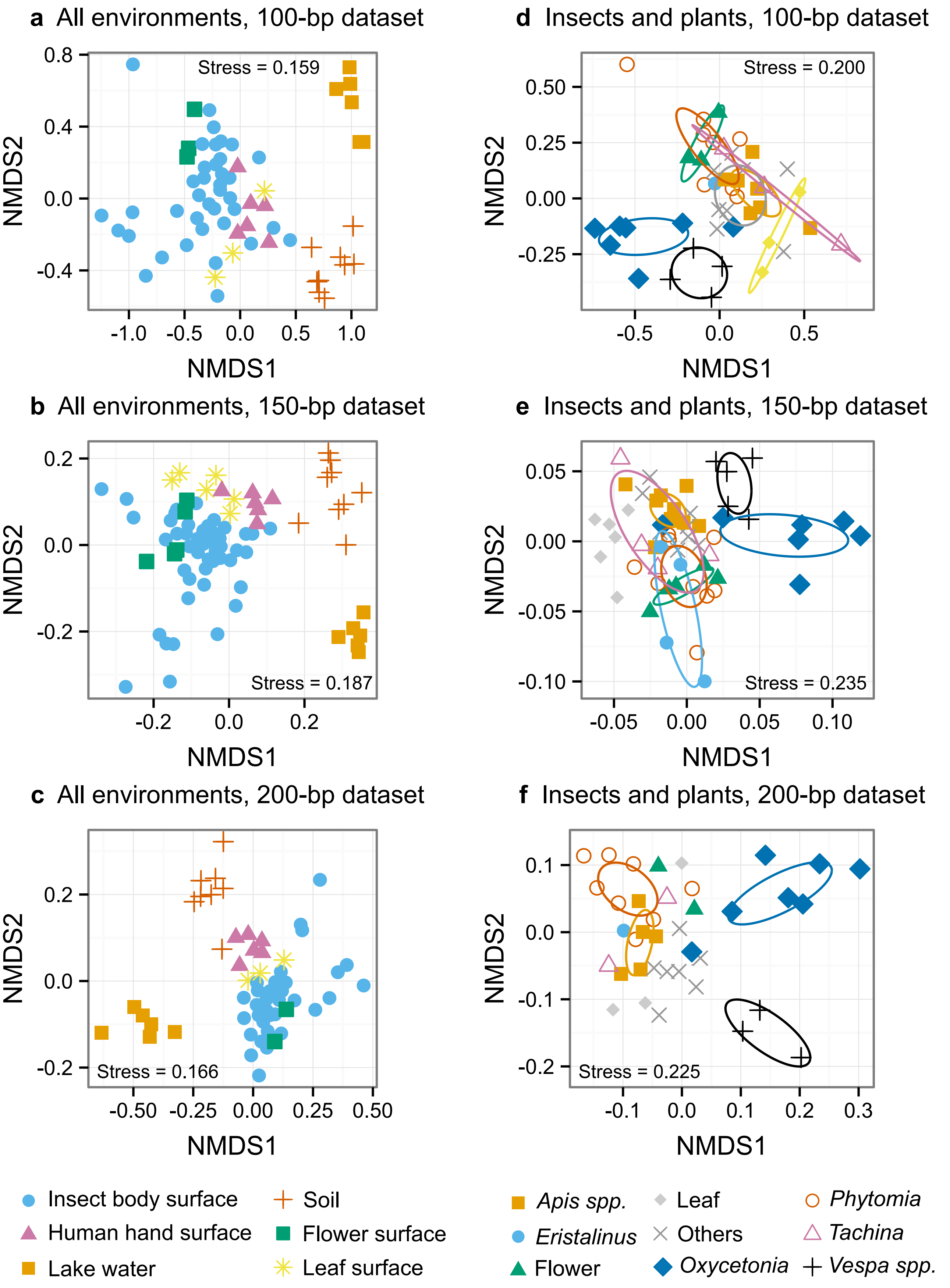

Figure S4

NMDS plots for the microbial communities recovered from insect and plant surfaces and other environments, using sequence datasets with (a, d) 100-bp, (b, e) 150-bp, and (c, f) 200-bp global trimming. These figures correspond to Figure 3 in the main text. NMDS plots for the 150-bp dataset are identical to those in Figure 3, but are shown here for direct comparison. Ovals in the figures in the right column (d–f) show 95% confidence intervals.
